# Fuzzy Model for Quantitative Assessment of Environmental Start-up Projects in Air Transport

**DOI:** 10.3390/ijerph16193585

**Published:** 2019-09-25

**Authors:** Miroslav Kelemen, Volodymyr Polishchuk, Beáta Gavurová, Stanislav Szabo, Róbert Rozenberg, Martin Gera, Jaroslaw Kozuba, Jakub Hospodka, Rudolf Andoga, Adriana Divoková, Peter Blišt’an

**Affiliations:** 1 Faculty of Aeronautics, Technical University of Kosice, Kosice 04121, Slovak Republic; 2 Faculty of Information Technologies, Uzhhorod National University, Uzhhorod 88000, Ukraine; volodymyr.polishchuk@uzhnu.edu.ua; 3 Research and Innovation Centre Bioinformatics, USP TECHNICOM, Technical University of Košice, Kosice 04001, Slovak Republic; beata.gavurova@tuke.sk; 4 Faculty of Aeronautics, Technical University of Kosice, Kosice 04121, Slovak Republic; stanislav.szabo@tuke.sk (S.S.); robert.rozenberg@tuke.sk (R.R.); rudolf.andoga@tuke.sk (R.A.); 5 Faculty of Mathematics, Physics and Informatics, Comenius University in Bratislava, Bratislava, Mlynská Dolina 84248, Slovak Republic; mgera@fmph.uniba.sk; 6 Faculty of Transport, Silesian University of Technology, Gliwice 44100, Poland; jaroslaw.kozuba@polsl.pl; 7 Faculty of Transport, Czech Technical University in Praque, Praque 16000, Czech Republic; xhospodka@fd.cvut.cz; 8 Faculty of Mechanical Engineering, Technical University of Kosice, Kosice 04121, Slovak Republic; adadivokova@gmail.com; 9 Faculty of Mining, Ecology, Process Control and Geotechnology, Technical University of Kosice, Kosice 04121, Slovak Republic; peter.blistan@tuke.sk

**Keywords:** environmental start-ups, development, assessment, air transport sector, a decision maker (DM), security of project financing

## Abstract

The purpose of this paper is to develop an applied fuzzy model of information technology to obtain quantitative estimates of environmental start-up projects in air transport. The developed model will become a useful tool for venture funds, business angels, or crowdfunding platforms for the development of innovative air transport businesses. Obtaining a quantitative estimate of the environmental start-up projects will increase the sustainability of the decision making on the security of financing of such projects by investors. This article develops a fuzzy evaluation model of project start-ups in air transport as an application of our neuro-fuzzy model in a specific air transport environment. The applied model provides output ranking of start-up project teams in air transport based on a four-layer neuro-fuzzy network. The presented model declares the possibilities of the application to solve these economic problems and offers the space for subsequent research focused on its usability in several areas of start-up development, in sectors and processes differentiated. The benefits are also visible for several types of policies, with an emphasis on decision-making processes in regulatory mechanisms to support the state funding in Slovakia, the EU etc.

## 1. Introduction

At present, the share of small and medium-sized enterprises in Slovakia is up to 99.9% of the total number of businesses while their significant socio-economic role is being associated with this share [1,2]. Small and medium-sized businesses generate job opportunities of almost 73.8% of the active workforce and share 53.6% of the added value [1]. According to the Slovak Business Agency [2], up to 96.9% of small and medium-sized enterprises are micro-enterprises. The sustainable economic growth and competitiveness of the economy is impossible without innovative development. The innovative development is linked to the activities of start-ups, which represent business initiatives with the high growth and innovation potential. Their role, in addition to long-term support for the smart and inclusive economic growth, should also attract foreign investments. From a procedural point of view, start-ups contribute to the development of sectors with the high added value, to the creation of regional and global competitiveness, as well as to the creation of employment of qualified labour force in Slovakia [3]. Slovakia has also built an image of an innovative economy through start-ups abroad. Each country tries to establish the most favourable legislative and regulatory environment by creating an appropriate ecosystem, as well as adequate financial schemes for supporting, in particular, the critical phases of start-ups [4]. It is also important to provide an access to non-financial tools. Slovakia also has a strategic material to sustain start-ups whose role is to support the process of creation and development of their ecosystem, as well as to support financing in the initial stages of their life. The issue of start-up financing is a strategic subject to permanent discussions in both professional and scientific circles. Our contribution reflects on these facts and solves the problem of looking for methods to create optimal decision-making mechanisms in the financing process of start-ups sector-oriented in the field of air transport with the aim of ensuring their financing security. There are several funding support models at present, the pressures for innovative development and the exploitation of the country’s innovation potential that create new forms, but a deeper analysis of funding processes with complementary risk or safety assessments are absent so far [5,6,7]. The main problem is their considerable methodological difficulty and dependence on expert evaluations. For this reason, mostly standard evaluation processes use the economic indicators that do not capture many non-financial, so-called soft, qualitative aspects of the riskiness of financing processes, and their contribution to decision-making mechanisms are thus very limited. The presented model stating the exact title mentioned in the analysis of the article) declares the application possibilities of solving these economic problems and offers space for subsequent research focused on its usability in several areas of start-up development, in sectors and processes differentiated. The secondary importance of our study, in supporting the creation of a decision-making platform enabling the creation of stabilization and regulatory mechanisms aimed at supporting the start-ups in Slovakia, is evident. New financial schemes will also require the availability of evaluation processes where the financial process efficiency and risk assessment at all stages will be compared which, in turn, may encourage the emergence and development of new hybrid forms of funding and new support programs. The benefits for many types of policies are also obvious. As part of government strategies for the start-up innovation and support, the Government of the Slovak Republic has planned to support financial literacy education, which could help transfer research knowledge into practice, as well as to maintain the participation of educational institutions in international programs stimulating cooperation with the private sector. This fact further underlines the importance of our study.

### 1.1. Overview of Domestic and Foreign Research Studies

Every day around the world, there are new start-up ideas that need to be improved: life, environment, safety, health, transport, education, etc., not only in air transport industry. A large number of environmental start-ups is emerging: to eliminate the negative impact of aviation; to improve the work of airlines, airports; for “green services” working around the air transport industry; to improve the air traffic control; to create the new means of air transport. 

Today, the number of companies in the aviation industry that are launching their own venture funds or crowdfunding platforms is constantly increasing. With the rapid development of technology, more and more large companies in the aviation industry believe that not all innovations begin inside. This confirms the creation of large aviation companies of subsidiary investment institutions to finance start-up projects in the air transport industry. In addition, there is a complex and urgent task to assess environmental start-up projects in air transport, the solution of which is of interest both to investment institutes and start-ups.

Start-ups have different stages of commercial development. The first stage is an output product on the market. After the successful completion of the first stage, the second stage comes—conquering the market as a competitive player in the industry. There is a large number of developed models [8,9] to estimate the amount of funding in the second stage since this stage is intended to finance an investment project that is on the market, and therefore quantitative estimates are used for the evaluation. Little attention is paid to the assessment of environmental start-ups at the stage of production of products for the market, especially in air transport. This raises the problem of the evaluation concept, since data on the unrealized project can only be expert-based, and therefore fuzzy. 

The analysis of the sources on this subject has resulted in the conclusion where the authors have introduced a number of evaluating start-up simulation and expert models using economic quantitative indicators. For example, a method of the index value of start-up setting out [10], a method of assessment of a start-up value [11], a model of assessment of start-ups by qualitative features [12] and others. Therefore, the environmental start-ups in air transport, discussed in the works can apply the general ideas and benefits of using fuzzy logic in decision support systems. [13,14]. A fuzzy set is used in the issue of start-up project evaluation, raised in the work [15], but it does not focus on the analysis of environmental start-ups in the air transport sector. In the work [16], there is a cognitive model for evaluating start-ups, but used only as an auxiliary tool for decision making by venture funds. Thus, the problems of evaluating environmental start-up projects in air transport, at the first stage of implementation with the use of the apparatus of fuzzy sets, did not rise.

In addition to evaluating the very start-up ideas for air transport systems, there is another feature. Investors are very cautious to finance such start-up projects because there is a problem of future customer confidence in the security of the implementation of such ideas in new air transport, either using technological innovations in existing transport or different systems and airport services or airlines. In such start-up projects, people can implement it to increase confidence because each project has a development team. The professionalism of developers of a start-up project depends on the success of its financing, as well as on increasing the credibility of consumers of the final product or technology. Therefore, people should develop, implement, and promote environmental start-up projects in air transport with professional experience and authority in the market of this industry.

In conclusion, after analysing the sources for this topic, there are no special models for evaluating the developers of environmental start-up projects in air transport. The authors have already raised the issue of the development of an information model of evaluation and output rating of start-up project development teams [17], but it is a formalized, general model. Therefore, at work [18] the task of informational modelling of the selection of a group of experts for various research objects has been solved, but possible indicators for the evaluation of environmental start-up developers in air transport have not been indicated. To do this, an applied informational neuro-fuzzy model for the elimination of the ranking of teams of environmental start-up project developers in air transport systems should improve. 

The selected theoretical framework has also been introduced in the study of authors as in [19] on the technology improving safety of crowdfunding platforms, or as in [20] on the security management education and training of critical infrastructure of sectors’ experts etc.

The problem of evaluating of start-ups can be formalized as a problem of decision making, which is commonly solved using different formalized methodologies like the multi-criteria decision making, expert systems, fuzzy inference systems or their combinations [21,22]. All these methodologies rely on the transfer of expert knowledge into a complex rule-base, however, the transfer of the expert knowledge is a heuristic process [23]. On the other hand, the mechanism of training neural networks does not rely on human expertise, but through a homogeneous structure of neural networks [24] it is difficult to extract the structured knowledge. Therefore, for this task it can be very beneficial to develop a unique form of neuro-fuzzy system that is combining the advantages of a well-structured knowledge base with the ability to objectively create this base using quantitative parameters (data).

The neuro-fuzzy networks have advantages over the multicriteria/expert methods. After receiving real data, the neuro-fuzzy networks can be trained, and their knowledge base could be supplemented. For the task, the rating of the developers’ team of an environmental start-up project in air transport, the neuro-fuzzy network develops and works with fuzzy expert input signals and, based on the knowledge base, displays adequate results. 

The plan is to compare at least three relevant methods for the objective and comprehensive solution of environmental start-up projects. The first part of the study focuses on innovative solutions using neuro-fuzzy systems for quantitative project evaluation and risk assessment. The second part of the study will use one of the multi-criteria decision-making methods. The third part of the study will be based on the use of a selected expert system. The knowledge gained in individual research questions will make it possible to compare relevant methods for these purposes and to formulate practical conclusions. The authors intend to use these conclusions for the evaluation of environmental start-up projects within the incubators of the University Science Park TECHNICOM in Kosice and at the Uzhhorod National University.

Construction of mathematical models, based on information about start-up projects and their developers, are subjective and inaccurate. It uses expertly generated information that reflects the substantive features of the researched object and is formulated in a natural language. In this case, the description of the object is a vague, qualitative reflection of decision-making knowledge. Therefore, it is advisable to use the fuzzy set theory to reflect knowledge of the object of study. The fuzzy model is a mathematical model based on the theory of fuzzy logic and fuzzy sets.

Application of this approach and the development of a fuzzy model of information technology for obtaining quantitative estimates of environmental start-up projects in air transport, in order to increase the safety of their financing, is the urgent task in the development of the innovative business.

### 1.2. Statement of the Assessment Problem

Let the investment institutions (venture funds, business angels, and capital investment organizations) evaluate some environmental start-ups of air transport projects$\;P=\left\{P_{1},P_{2},\ldots ,P_{n}\right\}$. 

The start-up (ideas) and its team of developers will evaluate each project:

$P_{k}=\left(S_{k};\;X_{k}\right),k=\bar{1,n}$, where $S=\left\{S_{1},S_{2},\ldots ,S_{n}\right\}$– is the plural environmental start-up in air transport projects, $X=\left\{X_{1},X_{2},\ldots ,X_{n}\right\}$—or the set of their developer teams. The incoming expert assessments of the proposed set of criteria will help evaluate start-up projects and teams. Without diminishing the universality, a single start-up project P will continue, in the case of a plurality of start-ups, on the initial estimates received. The mathematical model of the formulated problem is as follows:

(1) $$M\;\left(O_{S},\;\;O_{F}\right)=O_{P},$$

where $\left(O_{S},\;\;O_{F}\right)=P\left(S,\;\;X\right)$; $O_{S}$—fuzzy evaluation of the environmental start-up project S for air transport which is obtained by fuzzy multicriteria evaluation of alternatives; $O_{F}$—a rating team of developers; X an environmental start-up project for air transport which is obtained by the method of neuro-fuzzy networks; $O_{P}$—a quantitative aggregated initial estimate from the interval [0; 1]; M—an operator that matches the output variable $O_{P}$.

The researchers have used the system approach for an objective and integrated solution of the problem, based on multicriteria assessment alternatives for start-up projects [9,25] and neuro-fuzzy networks to evaluate their developers [26,27].

Thus, the purpose of this work is to develop a fuzzy model of information technology for obtaining quantitative estimates of environmental start-up projects in air transport in order to increase safety of their financing.

## 2. Materials and Methods 

### 2.1. Mathematical Model of the Fuzzy Estimation of Environmental Start-Ups of Air Transport Projects

The goal is to propose the following set of evaluation criteria $SK=\left(SK_{1},SK_{2},\ldots ,SK_{m}\right)$. To get an assessment for each criterion, the form of a question the authors used for a description of the corresponding assessment grading scale. It has been necessary to choose the variant for evaluation that is close to the truth [15]. 

$SK_{1}$—Proposes innovation is a technology or service for improvement of the environment in air transport:

1. Occurred at a given time (5 points);
2. Currently under development, with marketing and business plans (10 points);
3. At the stage of the working prototype, is tested by potential clients (15 points);
4. Currently receives income (20 points).

$SK_{2}$—The value of the environmental start-up for air transport:

1. Insignificance of novelty (5 points);
2. Makes travel more environmentally friendly, but does not solve any fundamental problems (10 points);
3. Solves a fundamental environmental problem in the air transport sector (15 points);
4. Will help preserves the environment and/or increase the environmental safety of air transport (i.e., an idea-based product is urgently needed on the market) (20 points).

$SK_{3}$—Strategic partners in the aviation industry:

1. Exchanged letters with potential partners not related to air transport (5 points);
2. An existing letter of intention prepared by a potential distributor for our product (10 points);
3. Several signed partnership agreements with aviation enterprises (15 points);
4. Partnership, licensing, delivery or sale agreements signed with many aviation enterprises (20 points).

$SK_{4}$—An intellectual property:

1. Patents have not yet been considered (0 points);
2. A preliminary application for a prepared and filed patent (5 points);
3. Team awaiting patents that have already been filed (10 points);
4. Availability of several patents that cover the entire chain of creation and commercialization of the invention, including trademarks (15 points).

$SK_{5}$—Presence of a specialist in the intellectual property:

1. The team will handle all intellectual property issues on its own (5 points);
2. A small company that does not have experience in working with air transport projects (10 points);
3. A small or medium-sized company that works with a large number of start-ups, air transport systems including (15 points);
4. An international law firm of intellectual property (20 points).

$SK_{6}$—Availability of a business plan for project implementation:

1. Does not exist (0 points);
2. Lots of errors occur (5 points);
3. Ideal in the opinion of developers (10 points);
4. Quite qualitative according to consultants, lawyers and accountants (15 points).

$SK_{7}$—Availability of sales and marketing plans:

1. No marketing plans (0 points);
2. Presence of the site with the product and popularization of it on the network (5 points);
3. The team has quality marketing and sale plans that include a combination of proven, cost-effective sales and marketing tactics (10 points).

$SK_{8}$—The environmental start-up project in air transport will compete with similar projects whose annual revenues make up:

1. Less than $100,000 (5 points);
2. $100,000–$500,000 (15 points);
3. More than $500,000 (20 points).

$SK_{9}$—Presence of a corporate lawyer:

1. A lawyer or a small firm that does not have experience in the investment field of air transport (5 points);
2. Medium-size company operating in the investment field of air transport (10 points);
3. A national law firm with a lot of connections in the venture community (15 points).

The set of criteria presented is open and investors can always add their own indicators when considering highly specialized air transport environmental projects. The given scale for the answers to the questions describes the level of the start-up. The higher the number of points, the more promising is the project.

The answers on the questions on the start-up project result in a set of numerical variables $SP=\left\{sp_{1},\;\;sp_{2},\ldots ,\;\;sp_{m}\right\}$ according to criteria $SK=\left\{SK_{1},\;\;SK_{2},\ldots ,\;\;SK_{m}\right\}$, taking values at a certain numerical interval. Each of these numerical variables, plural-carrier of the linguistic variable A, consists of the following terms: $A_{l1}$—“criterion evaluation $SK_{l}$ is much lower relative to the “investor's wishes”; $A_{l2}$—“criterion evaluation $SK_{l}$ is lower relative “investor’s wishes”; $A_{l3}$—“criterion evaluation $SK_{l}$ is close to the “investor's wishes”; $A_{l4}$—“criterion evaluation $SK_{l}$ is slightly better relative to the “investor's wishes”; $A_{l5}$—“criterion evaluation $SK_{l}$ is much better relative to the “investor's wishes”.

“Investor's wishes”—is a conditional convolution of points that satisfies the person who makes decisions when considering, evaluating and choosing start-ups. The model of fuzzy evaluation of the start-up projects follows in the next steps:

*Step 1*. Fuzzification of the input data

The resulting numerical variables $\left\{sp_{1},\;\;sp_{2},\ldots ,\;\;sp_{m}\right\}$ take different numerical values, then, for their comparison, it is necessary to have the normalized values. With this purpose, let us create the s—shaped membership function in the following form [17]:

(2) $$\mu_{l}\left(sp_{l},a_{l},b_{l}\right)=\{\begin{array}{cc} 0, & sp_{l}\leq a_{l};\\ 2{\left(\frac{sp_{l}-a_{l}}{b_{l}-a_{l}}\right)}^{2}, & a_{l}<sp_{l}\leq \frac{a_{l}+b_{l}}{2};\\ 1-2{\left(\frac{b_{l}-sp_{l}}{b_{l}-a_{l}}\right)}^{2}, & \frac{a_{l}+b_{l}}{2}<sp_{l}<b_{l};\\ 1, & sp_{l}\geq b_{l}. \end{array},$$

where $a_{l}$—minimal/$b_{l}$—the maximum number of points of the grading scale of evaluation by the criterion $SK_{l}$, $sp_{l}$—received the number of points on the grading scale for the considered start-up $\left(l=\bar{1,m}\right)$. 

*Step 2*. Take into account the wishes of a decision maker 

The decision maker (DM) is a person who makes the project evaluation decisions according to the criteria proposed by a group of start-up eco-system experts in the in the field of air transport.

Each DM criterion has its own reasoning, which should include the meaning of “investor’s wishes”. The vector $T=\left(t_{1},\;\;t_{2},\ldots ,\;t_{m}\right)$, according to the criteria $SK_{l}$,$\;\left(l=\bar{1,m}\right)$ will be denoted, and for each value, the membership function by the formula (2) will be calculated. The vector of the membership function “investor's wishes” is denoted by $\alpha =\left(\alpha_{1},\;\alpha_{2},\ldots ,\;\alpha_{m}\right),$ where $\alpha_{l}=\mu_{l\;}\left(t_{l}\right),$

*Step 3*. Estimation of the usefulness of the start-up

Regarding the “investor's wishes” and the results obtained for each criterion $SK_{l}$, project the value of the membership function on the plural of the linguistic variable carrier A. Each term A builds the membership function as follows (3–7) [15]:

(3) $$\mu_{A1}\left(\mu ;\alpha -\frac{\alpha }{2};\alpha -\frac{\alpha }{4}\right)=\{\begin{array}{cc} 1, & \mu \leq \alpha -\frac{\alpha }{2};\\ \frac{3\alpha -4\mu }{\alpha }, & \alpha -\frac{\alpha }{2}<\mu \leq \alpha -\frac{\alpha }{4}. \end{array},$$

(4) $$\mu_{A2}\left(\mu ;\alpha -\frac{\alpha }{2};\alpha -\frac{\alpha }{4};\alpha \right)=\{\begin{array}{cc} \frac{4\mu -2\alpha }{\alpha }, & \alpha -\frac{\alpha }{2}<\mu \leq \alpha -\frac{\alpha }{4};\\ \frac{4\alpha -4\mu }{\alpha }, & \alpha -\frac{\alpha }{4}<\mu \leq \alpha . \end{array},$$

(5) $$\mu_{A3}\left(\mu ;\alpha -\frac{\alpha }{4};\alpha ;\alpha +\frac{\alpha }{4}\right)=\{\begin{array}{cc} \frac{4\mu -3\alpha }{\alpha }, & \alpha -\frac{\alpha }{4}<\mu \leq \alpha ;\\ \frac{5\alpha -4\mu }{\alpha }, & \alpha <\mu \leq \alpha +\frac{\alpha }{4}. \end{array},$$

(6) $$\mu_{A4}\left(\mu ;\alpha ;\alpha +\frac{\alpha }{4};\alpha +\frac{\alpha }{2}\right)=\{\begin{array}{cc} \frac{4\mu -4\alpha }{\alpha }, & \alpha <\mu \leq \alpha +\frac{\alpha }{4};\\ \frac{6\alpha -4\mu }{\alpha }, & \alpha +\frac{\alpha }{4}<\mu \leq \alpha +\frac{\alpha }{2}. \end{array},$$

(7) $$\mu_{A5}\left(\mu ;\alpha +\frac{\alpha }{4};\alpha +\frac{\alpha }{2}\right)=\{\begin{array}{cc} \frac{4\mu -5\alpha }{\alpha }, & \alpha +\frac{\alpha }{4}<\mu \leq \alpha +\frac{\alpha }{2};\\ 1, & \mu \geq \alpha +\frac{\alpha }{2}. \end{array},$$

Depending on the interval μ it belongs in, for each criterion one or another membership function $\mu_{Alg}\;\left(g=\bar{1,5}\right)$, relative to “investor's wishes” α, is chosen. Then for each criterion $SK_{l}$, a linguistic meaning, and an assessment of the validity of the term is the result. The assessment of the criterion belongs to one or the other term. This gives the opportunity to reveal the subjectivity of the recruited expert points and to understand the presented start-up project [15].

*Step 4.* Quantitative evaluation of the project regarding DM wishes

Let the DM have own considerations regarding the choice of terms for the criteria $SK_{l}$. These terms, called desirable and designated, are $A{\;}_{lg}^{*}\;\left(l=\bar{1,m};\;g=\bar{1,5}\right)$. Then the estimates for the obtained and desired terms use the next membership function in the calculation (8) [15]:

(8) $$\mu \left(O_{l}\right)=\max\;\left\{\;\mu \;\left(A_{l}\right);\;\mu \;\left(B_{l}\right)\right\},$$

where $\mu (A_{l})=\{\begin{array}{cc} \mu_{A\mathrm{lg}}, & A_{\mathrm{lg}}=A_{\mathrm{lg}}^{*},\\ 0, & A_{\mathrm{lg}}\neq A_{\mathrm{lg}}^{*}. \end{array}$, and $\mu \left(B_{l}\right)=\{\begin{array}{cc} \frac{\mu_{Alg}}{2} & A_{l\left(g\pm 1\right)}=A_{lg}^{*}\\ 0, & A_{l\left(g\pm 1\right)}\neq A_{lg}^{*}\{ \end{array}$$\left(l=\bar{1,m}\right)$.

The resulting membership function shows how much the start-up project meets the wishes DM for each criterion. The constructed functions of the membership (3–7) have intersections, then obtain either one or two terms for the criteria with the same amount of reliability accordingly. Therefore, the built-up function of membership (8) for the next stage chooses the largest of them [15].

*Step 5*. Introduction of weight coefficients

DM sets weight coefficients for each criterion $(p_{1},p_{2},\ldots ,p_{m}),$ from the interval [1; 10]. Then one can define normalized weight coefficients for each group of criteria [26]:

(9) $$w_{l}=\frac{p_{l}}{\sum_{l=1}^{m}p_{l}},\;l=\bar{1,m};\;w_{l}\in \left[0,\;1\right],$$

*Step 6*. Construction of the quantitative assessment of the start-up project and linguistic interpretation

Let us consider one of the convolutions for building aggregated assessment [27]. For example, take the average of weighted convolution:

(10) $$O_{S}=\overset{m}{\underset{l=1}{\sum }}w_{l}\cdot \mu \left(O_{l}\right).,$$

Let us introduce the linguistic variable $ES\left(O_{S}\right)$ = “Evaluation of the Environmental Start-up of the Air Transport Project”. The multiplier for variable $O_{S}$ is an interval [0; 1], and a set of values is a term-set $ES=\left\{es_{1},\;\;es_{2},\ldots ,\;\;es_{5}\right\}$. The resulting value of the formula (10) is comparable to one of the term sets: $O_{S}\in$ (0.67; 1] – $es_{1}$ = “Assessment of the environmental start-up project in air transport is high”. $O_{S}\in$ (0.47; 0.67] – $es_{2}$ = “Assessment of the environmental start-up project in air transport is above average”. $O_{S}\in$ (0.36; 0.47] – $es_{3}$ = “Assessment of the environmental start-up project in air transport is average”. $O_{S}\in$ (0.21; 0.36] – $es_{4}$ = “Assessment of the environmental start-up project in air transport is low”. $O_{S}\in$ [0; 0.21] – $es_{5}$= “Assessment of the environmental start-up project in air transport is very low”. These limits, if necessary, investors can change. The developed model reduces the subjectivity of expert evaluations, shows the place of the environmental start-up project in air transport among others, and takes into account the DM wishes.

### 2.2. Informational Neuro-Fuzzy Model for Output Rating of Teams of the Environmental Start-Up Project in Air Transport 

**Figure 1:**
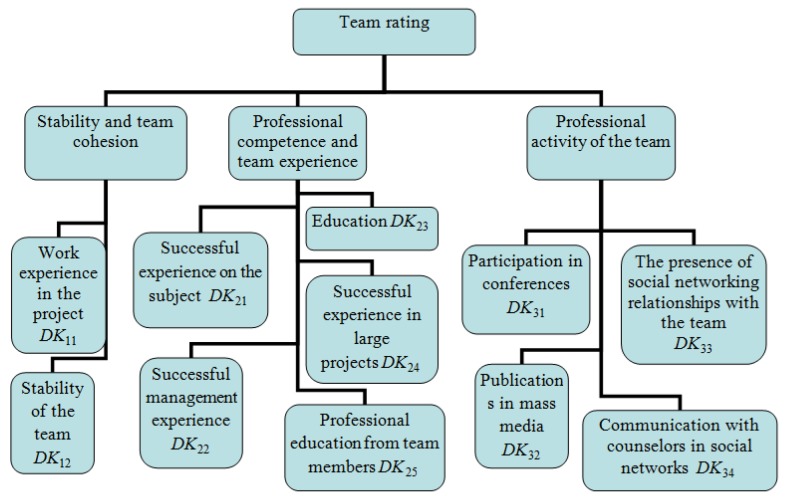
Scheme of evaluation criteria for teams of developers of environmental start-up projects.

Let the entrance of the neuro-fuzzy network provide expert data of the start-up team which has developed a project for air transport X according to the criteria of evaluation $DK=\left(DK_{11},\;\;DK_{12},\ldots ,\;\;DK_{34}\right)$. For this purpose, for example, the following set of evaluation criteria for a team of developers of environmental start-up projects create three groups, Figure 1. The evaluation criteria have the form of a questionnaire, where each team chooses the answer that comes close to them [17]. 

The first group of criteria is stability and team cohesion [17].

$DK_{11}$ – The length of work on the project is measured in months of work:

1. From 0 to 6 months;
2. From 6 to 12 months
3. From 12 to 24 months;
4. Months 24 and more.

$DK_{12}$ – Changing of leaders and team members determines the stability of the team:

1. Totally new team members and part of the leaders;
2. Insignificant changes in the number of team members;
3. The composition of the team is unchanged since all members and leaders meet the requirements of professionalism;
4. The team’s initial membership is unchanged, but there was an expansion of team members and leaders to reach the highest competence in the project.

Professional competence and team experience create the second group of criteria. 

$DK_{21}$ – Successful experience of leaders in air transport projects:

1. Experience is absent as the project is the first one;
2. Availability of the first experience in air transport and obtaining a small income;
3. A successful innovative air transport environmental project has been implemented;
4. Leaders have implemented not only one successful air transport environmental project.

$DK_{22}$—Successful experience in managing air transport environmental projects:

1. Management experience is absent as this project is the first one;
2. Management experience available, but insignificant;
3. Mid-level managers available in the field of air transport;
4. Managers of high level available in the field of air transport.

$DK_{23}$ – Education leaders:

1. No technical or managerial aviation education;
2. Graduated college, or university student of aviation or environment;
3. Completed higher aviation or environmental education;
4. At least one of the leaders has a scientific degree in the specialization of the project.

$DK_{24}$—Successful experience of team members in air transport environmental projects:

1. The team members´ experience is absent as this project is the first one;
2. The experience of team members is available, but not in air transport environmental projects;
3. Experience of team members in air transport environmental projects;
4. All team members have experience in large/successful air transport or environmental projects.

$DK_{25}$—Professional education of team members [17]:

1. Team members do not have special education to implement the project;
2. Some team members have special education to implement the project;
3. Most team members have special education to implement the project;
4. All team members have special education to implement the project.

The third group of criteria is the professional activity of the team. 

$DK_{31}$—Participation of the team in conferences, investment sessions, or specialized events in the field of air transport or environment:

1. Not involved in professional events for the project;
2. There is a single activity;
3. Existing activity;
4. Existing and systematic activity of advanced training.

$DK_{32}$—Publications in mass media or professional online sources for air transport projects:

1. There are no publications;
2. Information about the project and the team available, but mainly in social networks;
3. No single information about the project and the team;
4. Present and systematic activity of publications and popularization of the project.

$DK_{33}$—The presence of team ties in social networks and messengers:

1. No links;
2. Few, isolated links;
3. Wide circles of friends in different social networks;
4. Large activities with a significant number of subscribers.

$DK_{34}$—The presence of ties with advisors in the sphere of air transport/environment in social networks:

1. No links;
2. Few, isolated links;
3. Wide circles of friends in different social networks;
4. Large activities with a significant number of subscribers.

Each criterion, evaluated by the team of developers of the start-up projects, is evaluated professionally using one of the terms, the following term-set of linguistic variables L = {L; BA; A; H}, where: L—“A low-level indicator”; BA—“An indicator below average”; A—“An average level of the indicator”; H—“ A high level of the indicator”. Therefore, “A low-level indicator” is the first answer to the question, and the last answer, is “a high level of the indicator” [17]. 

For every assessment, the expert also puts the “confidence factor”d in assigning an assessment [19] from the interval (0; 1). For example, if the answer is not the one that corresponds to the developer team, then the metric d corrects the accuracy of the answer.

Therefore, the input signals present the form of linguistic terms and coefficients of expert confidence in their assignment.

Then, have a look at the object of the species EF=f(X) for which the connection “input X—output EF”can be filed in the form of a set of production rules of fuzzy knowledge base:

If ($DK_{11}$ = $\left(L_{11};\;d_{11}\right)$ (with weight $\alpha_{11}$) and $DK_{12}$ = $\left(L_{12};\;d_{12}\right)$ (with weight $\alpha_{12}$)) (with weight $\alpha_{1}$) also ($DK_{21}$ = $\left(L_{21};\;d_{21}\right)$ (with weight $\alpha_{21}$) and … and $DK_{25}$ = $\left(L_{25};\;d_{25}\right)$ (with weight $\alpha_{25}$)) (with weight $\alpha_{2}$) also ($DK_{31}$ = $\left(L_{31};\;d_{31}\right)$ (with weight $\alpha_{31}$) and … and $DK_{34}$ = $\left(L_{34};\;d_{34}\right)$ (with weight $\alpha_{34}$)) (with weight $\alpha_{3}$) then $EF=ef_{g},g=\bar{1,5}$.

Where $DK_{ij},i=\bar{1,3};j=\bar{1,5}$—a criterion of evaluation of the *i*-th group, *j*—a serial number of the rule in the group; $L_{ij}$ —a variable with the term-set L for the *j*-th group an indicator *i*; $d_{ij}$—“a confidence factor” expert on assigning a variable $L_{ij}$; $\left(L_{ij};d_{ij}\right)$ – grouped input data received from *k*-th start-up team by $DK_{ij}$ criterion; $\alpha_{11},\;\alpha_{12},\;\alpha_{21},\ldots ,\;\alpha_{25},\;\alpha_{31},\ldots ,\;\alpha_{34}$– synaptic weight criteria from the interval [1; b]; $\alpha_{1},\;\alpha_{2},\;\alpha_{3}$ – synaptic weight groups of criteria according to the interval [1; b]; $EF=\left\{ef_{1},\;\;ef_{2},\ldots ,\;\;ef_{5}\right\}$ – a linguistic interpretation of the rankings of the teams of developers of the start-up [10].

The scale of the output variable $EF=\left\{ef_{1},\;\;ef_{2},\ldots ,\;\;ef_{5}\right\}$ offers the following: $ef_{1}$ = “the rating of the team environmental start-up project for air transport is high”; $ef_{2}$ = “the rating of the team environmental start-up project for air transport is higher than the average”; $ef_{3}$ = “the rating of the team environmental start-up project for air transport is average”; $ef_{4}$ = “the rating of the team environmental start-up project for air transport is low”; $ef_{5}$ = “the rating of the team environmental start-up project for air transport is very low”.

**Figure 2:**
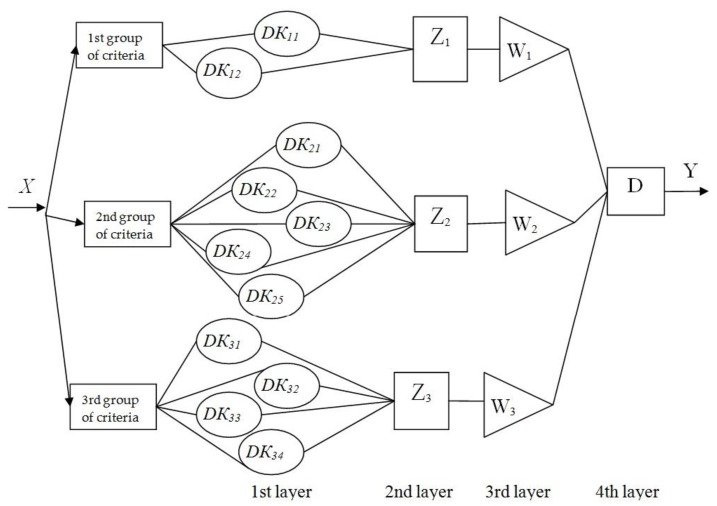
The structure of the neuro-fuzzy network of the team environmental start-up project for air transport.

The aggregated rating of developer team ratings can be presented in the form of a four-layer neuro-fuzzy network of type integrated neuro-fuzzy systems (similar to Mamdani neuro-fuzzy approximation) [24], Figure 2. 

Next, let us consider in more details what happens on each layer of the neuro-fuzzy network. 

1st layer

The fuzzification operation is performed in the first layer neurons, which means that for each input value (L; d) the value of the membership function acquires conformity μ (O). Therefore, it is necessary to establish membership rules at the first level in order to obtain a standardized estimate of the input data. Let the term-set of linguistic variables L = {L; BA; A; H} represent on a certain numerical interval $\left[a_{1};\;a_{5}\right]$ where $L\in \left[a_{1};\;a_{2}\right],$ $BA\in \left[a_{2};\;a_{3}\right],$ $A\in \left[a_{3};\;a_{4}\right],$ $H\in \left[a_{4};\;a_{5}\right]$. The value of breakdowns may be determined in the learning process of a neuro-fuzzy network using real data from teams of developers of environmental start-up projects for air transport. Let´s calculate criterion estimates O using linguistic variables L, “a confidence factor” expert on their assignment d and value decomposition interval $\left[a_{1};\;a_{5}\right]$, with the help of a characteristic function [17]:

(11) $$O_{ij}=\{\begin{array}{ccc} a_{2}\cdot d_{ij}, & if & L_{ij}\in L;\\ a_{3}\cdot d_{ij}, & if & L_{ij}\in BA;\\ a_{4}\cdot d_{ij}, & if & L_{ij}\in A;\\ a_{5}\cdot d_{ij}, & if & L_{ij}\in H. \end{array},$$

This will make it possible to adjust the assessment regarding the expert's confidence in the assignment, or reckon how close is the answer to the question of the developer team to the truth. Next, the membership rule to help S -a similar membership function as follows [17,26]:

(12) $$\mu \left(O_{ij}\right)=\{\begin{array}{cc} 0, & O_{ij}\leq a_{1};\\ 2{\left(\frac{O_{ij}-a_{1}}{a_{5}-a_{1}}\right)}^{2}, & a_{1}<O_{ij}\leq \frac{a_{1}+a_{5}}{2};\\ 1-2{\left(\frac{a_{5}-O_{ij}}{a_{5}-a_{1}}\right)}^{2}, & \frac{a_{1}+a_{5}}{2}<O_{ij}<a_{5};\\ 1, & O_{ij}\geq a_{5}. \end{array},$$

Constructed in this way, it is clear from the membership function, the resulting value will go to 1, in case if there is the high estimation of the criterion and the sufficiently high confidence of the expert on the assignment [10]. Thus, the experts' evaluations of teams of developers of start-up projects and expert confidence in their assignment turn to normalized comparable data [9,25]. 

Consequently, the subjectivity of expert opinions discloses and switches from fuzzy expert linguistic evaluations to normalized and comparable in neurons of the first layer. 

2nd layer

On the second layer, the authors have grouped the calculation of functions of postsynaptic potential according to the criteria of evaluation. The second layer contains the number of neurons that corresponds to the number of groups of criteria. Let the DM set the synaptic weights $\alpha_{11},\alpha_{12},\alpha_{21},\ldots ,\alpha_{25},\alpha_{31},\ldots ,\alpha_{34},$ from the interval [1;b] for each criterion. Input signals with synaptic weights form the value of the excitatory level of the neurons $Z_{1},Z_{2},Z_{3}$. The calculation of the postsynaptic potential functions is as follows:

(13) $$Z_{1}=\frac{1}{\alpha_{11}+\alpha_{12}}\cdot \left(\mu \left(O_{11}\right)\cdot \alpha_{11}+\mu \left(O_{12}\right)\cdot \alpha_{12}\right),$$

(14) $$Z_{2}=\frac{1}{\alpha_{21}+\alpha_{22}+\ldots +\alpha_{25}}\cdot \left(\mu \left(O_{21}\right)\cdot \alpha_{21}+\mu \left(O_{22}\right)\cdot \alpha_{22}+\ldots +\mu \left(O_{25}\right)\cdot \alpha_{25}\right),$$

(15) $$Z_{3}=\frac{1}{\alpha_{31}+\alpha_{32}+\ldots +\alpha_{34}}\cdot \left(\mu \left(O_{31}\right)\cdot \alpha_{31}+\mu \left(O_{32}\right)\cdot \alpha_{32}+\ldots +\mu \left(O_{34}\right)\cdot \alpha_{34}\right).$$

Output neurons of the second layer $Z_{1},\;Z_{2},\;Z_{3}$, normalized because the calculations use the relative importance of the synaptic scales of the criteria [17].

3rd layer

On the third layer, there is the second layer correction of neurons, in relation to the importance of one or the other group of evaluation criteria, provided. In this case, for each group of criteria, the person who is making the decision has his own considerations regarding the synaptic weights $\alpha_{1},\;\alpha_{2},\;\;\alpha_{3}\;$respectively, from an interval [1; b]. The functions of the postsynaptic potential of the third layer of neurons will be calculated in the following way [17]:

(16) $$W_{i}=\frac{\alpha_{i}}{\sum_{i=1}^{3}\alpha_{i}}\cdot Z_{i},i=\bar{1,3},$$

Similarly, the output neurons of the third layer $W_{1}$, $W_{2}$, $W_{3}$ will be normalized since the calculations use the relative importance of the synaptic weights of the groups of criteria.

4th layer

On the fourth layer, the data will be defuzzificated. To do this, the following activation function in the output neuron should be is used [17]:

(17) $$O_{F}=\overset{3}{\underset{i=1}{\sum }}W_{i},$$

The method of creating the knowledge base and generating new rules of production is a proposal for training the neuro-fuzzy network. Our previous article provides a description of the training algorithm [17]. The network training was conducted on a training set of data from a university team of developers (28 teams) and the work was verified on the basis of on test data of successful start-up projects and their developers [17]. Rating levels for start-up teams of developers in air transport systems are the result of the training on neuro-fuzzy networks. Let us match the aggregated score $O_{F}$ with output variable $EF=\left\{ef_{1},\;\;ef_{2},\ldots ,\;\;ef_{5}\right\}$ as follows: $O_{F}\in$ (0,87; 1] – $ef_{1}$; $O_{F}\in$ (0,67; 0,87] – $ef_{2}$; $O_{F}\in$ (0,37; 0,67] – $ef_{3}$; $O_{F}\in$ (0,21; 0,37] – $ef_{4}$; $O_{F}\in$ [0; 0,21] – $ef_{5}$. 

### 2.3. Determination of the Quantification for the Withdrawal of the Security Rating of Project Financing

The assessment of the start-up project $O_{S}$, and evaluation of the team implementation $O_{F}$, is a result of the evaluation, for a submitted environmental start-up P in air transport. In addition to the quantitative evaluation, a linguistic interpretation ES and EF is obtained. In the case of a plurality of projects, the following data are acquired, Table 1. 

Based on estimation$\;O_{S}$, $O_{F}$, a model for obtaining a quantitative aggregate initial estimation $O_{P}$ from the interval [0; 1] is a proposal for the considered start-up of the project. To do this, a convolution is used in the form of the Gaussian two-dimensional functions [28], on the interval [0; 1] by coordinates *x, y, z*, according to the following formula:

(18) $$O_{P}=F\left(O_{S};O_{F}\right)=e^{-\left({\left(O_{S}-1\right)}^{2}+\left(O_{S}-1\right)\left(O_{F}-1\right)+{\left(O_{F}-1\right)}^{2}\right)},$$

**Figure 3:**
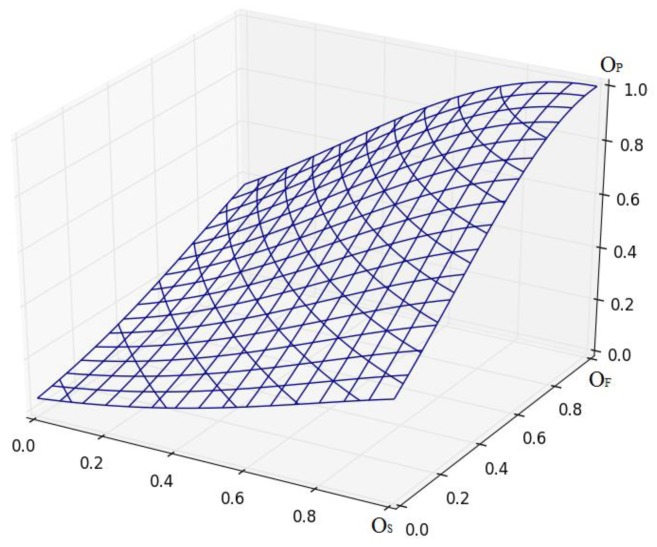
Graphic interpretation of aggregated estimation.

Figure 3 presents the graphical interpretation of aggregated estimation.

Thus, we obtain an initial estimate $O_{P}\;$from the interval [0; 1]. 

In order to determine the level of security of financing, the environmental start-up of projects in air transport received value according to the formula (18) compared to one of the term settings $PS=\left\{ps_{1},\;\;ps_{2},\ldots ,\;\;ps_{5}\right\}$ with the following content:

- $O_{P}\in$ (0.7; 1] – $ps_{1}$ = “a high level of security of environmental start-up project financing in air transport”. It’s the highest level of security financing and provides very low expectations regarding the risks of non-compliance with project development obligations. The team’s ability to react promptly and solve current or strategic problems in project implementation is very high;
- $O_{P}\in$ (0.5; 0.7] – $ps_{2}$ = “a security level of financing of an environmental start-up project in air transport above average”. It is a high level of security financing and provides low expectations of non-compliance with project development obligations. The ability of the team to react in a timely manner and to solve current or strategic problems of the project implementation is low, but this ability can reduce negative operational or economic changes;
- $O_{P}\in$ (0.4; 0.5] – $ps_{3}$ = “Van average safety level of environmental start-up project financing in air transport”. It is a speculative level of security financing. There is the possibility of developing project risks and / or conflict risks in the middle of the team due to the deterioration of economic changes;
- $O_{P}\in$ (0.2; 0.4] – $ps_{4}$= “a low level of security of environmental start-up project financing in air transport”. The rating says that realizing the project in time is not a real opportunity. The ability of a team to work depends on the favourable business and economic conditions;
- $O_{P}\in$ [0; 0.2] – $ps_{5}$ = “a very low level of security of environmental start-up project financing in air transport”. Very high risks of non-fulfillment of project development obligations and the formed start-up team are not able to work on the project.

## 3. Results

We will test the results of the paper on an example of the security of the financing of the environmental start-up projects in air transport. Let us have five environmental start-ups projects in air transport $P=\left\{P_{1},\;P_{2},\ldots ,\;P_{5}\right\}$ to evaluate the security of their financing by investors. Each project will be evaluated by the start-up (ideas) and the team of developers: $P_{k}=\left(S_{k};\;X_{k}\right),\;\;k=\bar{1,5}$. The authors could take all the projects, under consideration, from the university incubators (the Technical University of Kosice—TECHNICOM and the Uzhhorod National University). The projects have undergone an expert evaluation on the proposed sets of criteria. The input data for the project, weighting factors of the criteria, "investor's wishes“ T and wish the meaning of the term DM is given in Table 2. Expert evaluation data, teams presented in Table 3.

First, we will evaluate start-up projects for the fuzzy evaluation model, according to the steps below:

*Steps 1–2*. The fuzzification of the input data and the consideration of the DM require calculating the value of membership functions according to Equation (2), Table 4.

*Step 3*. Estimation of the usefulness of the start-up, for each criterion, we obtain the linguistic meaning and validity of the term for (3–7), Table 5.

*Steps 4–5*. The quantitative evaluation of the project in terms of DM´s wishes. The result of computing the estimates for the obtained and desirable terms using the membership function (8) and determining the normalized weight coefficients for (9), are presented in Table 6.

*Step 6*. We build a quantitative estimate of the environmental start-up of the projects by the formula (10): $O_{S}=$ (0.095; 0.263; 0.218; 0.516; 0.528). As you can see, the best start-up project $S_{5}$, with linguistic interpretation – $es_{2}$ = “an assessment of an environmental start-up project in air transport is above average”.

Then, the evaluation of development teams continued with the fuzzification of the input signals in the neurons of the first layer. To achieve this, the membership function in a numerical interval [0; 10], where L∈[0;2], BA∈[2;5], A∈[5;8], H∈[8;10] must be defined. We use equations (11–12) to obtain the value of the membership function, and we write the results in Table 7.

On the second, up to third layer we calculate the functions of postsynaptic potential. Let the DM´s wishes be for the synaptic scales of the criteria (8; 9; 8; 10; 9; 10; 7; 8; 6; 7; 9)∈[1;10] and the synaptic weights for a group of criteria (10; 9; 8)∈[1;10]. By the equations (13–15) for the second layer and (16) for third layer, the results follow in Table 8.

Next, in the fourth layer, there is the defuzzification of the data for (17): $O_{F}=$ (0.234; 0.492; 0.445; 0.749; 0.746). As you can see, the best developer – $X_{4}$, with linguistic interpretation – $ef_{2}$ = “the rating of the team environmental start-up project for air transport is higher than the average”.

Based on ratings $O_{S}$, $O_{F}$ the quantitative aggregate initial estimates $O_{P}\;$is received from the interval by the formula (18): $O_{P_{1}}=$ (0.095; 0.234) = 0.123; $O_{P_{2}}=\;$ 0.309; $O_{P_{3}}=$ 0.258; $O_{P_{4}}=$ 0.658; $O_{P_{5}}=$ 0.665. The projects can be ranked according to the quantified estimates: $P_{5};$ $P_{4};$ $P_{2};$ $P_{3};$ $P_{1}$.

Estimates suggest that the conclusion means the best combination of the environmental start-up and its development teams in air transport—$P_{5}$, and “the level of security of funding for the environmental start-up project in air transport is above average”.

## 4. Discussion

The applied fuzzy model of information technology for quantitative estimation of the environmental start-up projects in air transport will increase the degree of validity of decision making regarding the safety of such project financing by investors. For this purpose, a mathematical model of fuzzy evaluation of the environmental start-up of the air transport project and an informative neuro-fuzzy model for the elimination of the rating of developer teams of environmental start-up projects for air transport have developed. Based on the initial estimates for the project start-up, there is a model for obtaining a quantitative aggregate estimate. The assessment has reached a decision on the security of project financing. The developed model of the fuzzy estimation of the environmental start-up of air transport projects has a number of advantages. First, it gives an opportunity to understand the nature and place of the proposed project start-up in the estimation area. It then reveals the uncertainty of upcoming expert assessments using a set of linguistic variables in relation to the " the investor´s wish", and determines the level of start-up assessment and its linguistic value taking into account the wishes of DM in considering, evaluating and selecting start-ups for air transport. The disadvantages of this approach include the use of different models of membership functions, which can lead to ambiguity of the results [15]. There are some advantages of the informational neuro-fuzzy model for eliminating the rating of environmental start-up teams of developers for air transport. The objectivity of expert assessments in evaluating teams of developers for air transport has increased. A neuro-fuzzy network which has the ability to change the the synaptic weighting of criteria and groups of criteria for evaluating the teams of developers for air transport is, fundamental. The arrival of experimental data help conduct the neuro-fuzzy network training by completing the knowledge base and adjusting the rating levels of the developer teams of environmental start-up projects. 

The disadvantages of this approach contribute to the fact that the received membership function in the neuro-fuzzy network corresponds to the stage of rough debugging. Therefore, the process of debugging a neuro-fuzzy network, depending on the breakdown of the gap $\left[a_{1};a_{5}\right]$ is possible in a sample of reliable experimental data [17].

The result of the paper is an applied model of information technology for obtaining a quantitative assessment of environmental start-up projects in air transport, working with fuzzy data, and increasing the validity of the decision-making process regarding the security of their financing. Its output is a quantitative assessment and a linguistic interpretation of the level of security of the project environmental start-up financing in air transport. The rationality of this assessment proves the benefits of the developed model. The correct use of the apparatus of fuzzy logic, fuzzy sets, and neuro-fuzzy networks, confirmed by the research results, ensures the reliability of the obtained results. The findings of this study also extend the authors´ research in the selected security and environmental aspects in the aviation sector, as the authors in [29], or as in the work on the flight planning and its impact on the environment [30], or as in [31] on the selected forecast methods, or in the work on the non-stationary noise analysis [32] etc. They will be the starting point for the following research on the environmental projects risk assessment model. 

The following study of the problems will continue in approbation of the developed fuzzy model for a wide selection of start-ups in the field of air transport, the development of training methods for this technology and the creation of software. The results of this study (or these analyses) also bring several methodological benefits. The application of the presented model reveals several possibilities for fine-tuning evaluation criteria (Figure 1) and offers application space for the use of other complementary expert methods. E.g. the structural analysis of Matriced’ Impacts Croises Multiplication Appliquee a un Classement (MIC-MAC, known in English as the Cross-Impact Matrix), the advantage of which is that it also takes into account indirect, i.e., mediated relationships between variables [33]. The result is their distribution according to the degree of influence and dependence. In this way, it would be possible to investigate experimentally the complex functionality of the start-up financing system linking the presented model use [34,35]. Likewise, the application of the Analytical Hierarchy Process (AHP) would support processes for selecting optimal evaluation criteria, especially in situations where there are multiple stakeholders and some heterogeneity of preferred parameters, whether input or output. The interconnection of the above-mentioned expert methods of MIC-MAC and AHP would be particularly beneficial in the preparatory phases of using the presented model, and would encourage further experimental development of methodologies, the application of which is increasingly desirable in decision-making and evaluation mechanisms.

## 5. Conclusions

The real research task of obtaining a quantitative estimation of environmental start-up projects in air transport is to increase the safety of their funding. The model applied has helped develop the quantitative estimating environmental start-up projects in the field of air transport at the stage of product output to the market, in the conditions of uncertainty with the use of the apparatus of fuzzy mathematics and create a model for evaluating and eliminating the environmental start-up team rating using a neuro-fuzzy network. The following results are as follows:

- Formulations of a set of nine criteria for estimating the environmental start-up of air transport projects and a grading scale of assessments, in the form of a question-answer to obtain an assessment for each criterion. In order to evaluate teams of developers of the start-up projects in the air transport system, the authors have classified eleven proposed criteria in three groups and presented the input data in the form of four linguistic terms and the expert confidence coefficient for their assignment;
- An applied model of fuzzy evaluation of air transport environmental project start-ups, based on six steps, allows reducing the subjectivity of expert assessments by valuing the input data of the evaluation grading scale. The features of the model allow revealing the essence and place of the project start-up among others. The base includes the "investor's wishes" and the results obtained for each criterion, projecting the value of the function of belonging to the plural of the carrier of the linguistic variable. Taking into account the wishes of the DM, the model determines the level of assessment of the environmental start-up of the project in air transport;
- The research team has developed the applied informational neuro-fuzzy model for the elimination of the rating of environmental start-up developer teams for air transport, based on four-layer neural-fuzzy network. The model reveals the subjectivity of expert opinions for outputting the rating of the development teams and does not require much computation, formulated production rules of the fuzzy knowledge base, and five levels of the rating of teams of developers for air transport;
- Based on the conducted research, the model of quantitative aggregate initial estimation was proposed using a two-dimensional Gaussian function for the aggregation of results. The levels were set for the financing of environmental start-up projects in air transport for the security;
- The developed applied fuzzy model is tested on an example of the financing security of environmental start-up projects in air transport.

Creation of technology for obtaining a quantitative assessment, launching air transport environmental projects to improve the security of their funding, based on the developed applied fuzzy model, will be a necessary tool for investment institutions (venture funds, “business angels”, crowdfunding platforms) in developing innovative air transport business and promoting funding for such projects. This will provide significant support for the creation and development of new financial schemes aimed at financing the critical phases of start-ups that are essential to exploit the maximum potential of start-ups while creating a favourable legislative and regulatory framework and accessing non-financial tools.

The problem of the impact of aviation on our public health in the context of the United Nations Agenda for the Sustainable Development (2030), which supports the spirit of the Sustainable Development Goals focused on the Goal 13 Climate Action too, requires the effective solutions. The proposals of environmental projects in the aviation sector are an important source of innovation and therefore the authors are exploring the new tools and methodology for their assessment, which was the aim of the paper.

## Figures and Tables

**Table 1:** Data for the case of a plurality of projects.

Projects	Start-up Projects		Team Developers	
	Estimation	Linguistic Interpretation	Estimation	Linguistic Interpretation
$P_{1}$	$O_{S_{1}}$	$ES_{1}$	$O_{F_{1}}$	$EF_{1}$
$P_{2}$	$O_{S_{2}}$	$ES_{2}$	$O_{F_{2}}$	$EF_{2}$
…	…	…	…	…
$P_{n}$	$O_{S_{n}}$	$ES_{n}$	$O_{F_{n}}$	$EF_{n}$

**Table 2:** Input of environmental start-up projects on the criteria of evaluation.

Name Criteria	p	T	$A{\;}_{lg}^{*}{\;}^{1}$	$S_{1}$	$S_{2}$	$S_{3}$	$S_{4}$	$S_{5}$
$SK_{1}$	10	15	$A{\;}_{13}^{*}$	5	5	10	20	20
$SK_{2}$	10	18	$A{\;}_{23}^{*}$	5	20	5	20	15
$SK_{3}$	7	20	$A{\;}_{34}^{*}$	10	5	20	10	20
$SK_{4}$	7	10	$A{\;}_{42}^{*}$	0	5	0	15	15
$SK_{5}$	6	15	$A{\;}_{53}^{*}$	10	10	15	20	20
$SK_{6}$	8	15	$A{\;}_{63}^{*}$	0	10	5	15	15
$SK_{7}$	7	10	$A{\;}_{73}^{*}$	5	5	5	10	10
$SK_{8}$	9	18	$A{\;}_{83}^{*}$	5	5	5	15	15
$SK_{9}$	6	13	$A{\;}_{92}^{*}$	5	5	10	10	15

^1^ These terms, called desirable.

**Table 3:** Inputs of developers of environmental start-up projects on the criteria of evaluation.

Name Criteria	$X_{1}$		$X_{2}$		$X_{3}$		$X_{4}$		$X_{5}$	
	L	d	L	d	L	d	L	d	L	d
$DK_{11}$	L	0.6	L	0.9	A	0.8	H	0.9	H	0.7
$DK_{12}$	BA	0.7	BA	0.8	A	0.6	A	0.8	H	0.9
$DK_{21}$	L	0.8	BA	0.7	A	0.4	H	0.7	H	0.7
$DK_{22}$	BA	0.8	A	0.9	BA	0.8	H	0.7	A	0.8
$DK_{23}$	BA	0.6	A	0.8	BA	0.6	H	0.9	A	0.6
$DK_{24}$	BA	0.6	BA	0.9	A	0.8	A	0.8	A	0.5
$DK_{25}$	A	0.8	A	0.7	BA	0.8	A	0.7	A	0.7
$DK_{31}$	A	0.9	H	0.8	BA	0.8	BA	0.9	BA	0.8
$DK_{32}$	L	0.8	H	0.6	L	0.8	H	0.9	H	0.9
$DK_{33}$	L	0.7	H	0.6	H	0.6	H	0.6	H	0.9
$DK_{34}$	BA	0.6	A	0.8	BA	0.8	BA	0.8	A	0.8

**Table 4:** Normalized data on the criteria for evaluating environmental start-up projects.

Name Criteria	$S_{1}$	$S_{2}$	$S_{3}$	$S_{4}$	$S_{5}$	α
$SK_{1}$	0.000	0.000	0.222	1.000	1.000	0.778
$SK_{2}$	0.000	1.000	0.000	1.000	0.778	0.964
$SK_{3}$	0.222	0.000	1.000	0.222	1.000	0.778
$SK_{4}$	0.000	0.222	0.000	1.000	1.000	0.778
$SK_{5}$	0.222	0.222	0.778	1.000	1.000	0.778
$SK_{6}$	0.000	0.778	0.222	1.000	1.000	1.000
$SK_{7}$	0.500	0.500	0.500	1.000	1.000	1.000
$SK_{8}$	0.000	0.000	0.000	0.778	0.778	0.964
$SK_{9}$	0.000	0.000	0.500	0.500	1.000	0.920

**Table 5:** Evaluating the usefulness of the start-up.

Name Criteria	$S_{1}$		$S_{2}$		$S_{3}$		$S_{4}$		$S_{5}$	
	$A_{lg}$	$\mu_{Alg}$	$A_{lg}$	$\mu_{Alg}$	$A_{lg}$	$\mu_{Alg}$	$A_{lg}$	$\mu_{Alg}$	$A_{lg}$	$\mu_{Alg}$
$SK_{1}$	$A_{11}$	1	$A_{11}$	1	$A_{11}$	1	$A_{14}$ $A_{15}$	0.857 0.143	$A_{14}$ $A_{15}$	0.857 0.143
$SK_{2}$	$A_{21}$	1	$A_{23}$ $A_{24}$	0.853 0.147	$A_{21}$	1	$A_{23}$ $A_{24}$	0.853 0.147	$A_{22}$ $A_{23}$	0.774 0.226
$SK_{3}$	$A_{31}$	1	$A_{31}$	1	$A_{34}$ $A_{35}$	0.857 0.143	$A_{31}$	1	$A_{34}$ $A_{35}$	0.857 0.143
$SK_{4}$	$A_{41}$	1	$A_{41}$	1	$A_{41}$	1	$A_{44}$ $A_{45}$	0.853 0.147	$A_{44}$ $A_{45}$	0.853 0.147
$SK_{5}$	$A_{51}$	1	$A_{51}$	1	$A_{53}$	1	$A_{54}$ $A_{55}$	0.853 0.147	$A_{54}$ $A_{55}$	0.853 0.147
$SK_{6}$	$A_{61}$	1	$A_{62}$ $A_{63}$	0.889 0.111	$A_{61}$	1	$A_{63}$	1	$A_{63}$	1
$SK_{7}$	$A_{71}$	1	$A_{71}$	1	$A_{71}$	1	$A_{73}$	1	$A_{73}$	1
$SK_{8}$	$A_{81}$	1	$A_{81}$	1	$A_{81}$	1	$A_{82}$ $A_{83}$	0.774 0.226	$A_{82}$ $A_{83}$	0.774 0.226
$SK_{9}$	$A_{91}$	1	$A_{91}$	1	$A_{91}$ $A_{92}$	0.826 0.174	$A_{91}$ $A_{92}$	0.826 0.174	$A_{93}$ $A_{94}$	0.652 0.348

**Table 6:** Values of assessments by evaluation criteria.

Name Criteria	$S_{1}$	$S_{2}$	$S_{3}$	$S_{4}$	$S_{5}$	w
$SK_{1}$	0	0	0	0.429	0.429	0.14
$SK_{2}$	0	0.853	0	0.853	0.387	0.14
$SK_{3}$	0	0	0.857	0	0.857	0.10
$SK_{4}$	0.5	0.5	0.5	0	0	0.10
$SK_{5}$	0	0	0.5	0.429	0.429	0.09
$SK_{6}$	0	0.445	0	1	1	0.11
$SK_{7}$	0	0	0	1	1	0.10
$SK_{8}$	0	0	0	0.387	0.387	0.13
$SK_{9}$	0.5	0.5	0.413	0.413	0.326	0.09

**Table 7:** Fuzzification of the input signals.

Name Criteria	$X_{1}$	$X_{2}$	$X_{3}$	$X_{4}$	$X_{5}$
$DK_{11}$	0.029	0.065	0.741	0.980	0.820
$DK_{12}$	0.245	0.320	0.461	0.741	0.980
$DK_{21}$	0.051	0.245	0.205	0.820	0.820
$DK_{22}$	0.320	0.843	0.320	0.820	0.741
$DK_{23}$	0.180	0.741	0.180	0.980	0.461
$DK_{24}$	0.180	0.405	0.741	0.741	0.320
$DK_{25}$	0.741	0.613	0.320	0.613	0.613
$DK_{31}$	0.843	0.920	0.320	0.405	0.320
$DK_{32}$	0.051	0.680	0.051	0.980	0.980
$DK_{33}$	0.039	0.680	0.680	0.680	0.980
$DK_{34}$	0.180	0.741	0.320	0.320	0.741

**Table 8:** Function of postsynaptic potential of neurons of the second and third layers.

Name Criteria	$X_{1}$	$X_{2}$	$X_{3}$	$X_{4}$	$X_{5}$
$Z_{1}$	0.143	0.200	0.593	0.853	0.905
$Z_{2}$	0.278	0.577	0.366	0.802	0.582
$Z_{3}$	0.298	0.762	0.350	0.559	0.732
$W_{1}$	0.053	0.074	0.219	0.316	0.335
$W_{2}$	0.093	0.192	0.122	0.267	0.194
$W_{3}$	0.088	0.226	0.104	0.166	0.217
