# Correction: Fuzzy Model for Quantitative Assessment of Environmental Start-up Projects in Air Transport. *Int. J. Environ. Res. Public Health* 2019, *16*(19), 3585

**DOI:** 10.3390/ijerph16245011

**Published:** 2019-12-09

**Authors:** Miroslav Kelemen, Volodymyr Polishchuk, Beáta Gavurová, Stanislav Szabo, Róbert Rozenberg, Martin Gera, Jaroslaw Kozuba, Rudolf Andoga, Adriana Divoková, Peter Blišťan

**Affiliations:** 1 Faculty of Aeronautics, Technical University of Kosice, Kosice 04121, Slovak Republic; stanislav.szabo@tuke.sk (S.S.); robert.rozenberg@tuke.sk (R.R.); rudolf.andoga@tuke.sk (R.A.); 2 Faculty of Information Technologies, Uzhhorod National University, Uzhhorod 88000, Ukraine; volodymyr.polishchuk@uzhnu.edu.ua; 3 Research and Innovation Centre Bioinformatics, USP TECHNICOM, Technical University of Košice, Kosice 04001, Slovak Republic; beata.gavurova@tuke.sk; 4 Faculty of Mathematics, Physics and Informatics, Comenius University, Bratislava, Mlynská Dolina 84248, Slovak Republic; mgera@fmph.uniba.sk; 5 Faculty of Transport, Silesian University of Technology, Gliwice 44100, Poland; jaroslaw.kozuba@polsl.pl; 6 Faculty of Mechanical Engineering, Technical University of Kosice, Kosice 04121, Slovak Republic; adadivokova@gmail.com; 7 Faculty of Mining, Ecology, Process Control and Geotechnology, Technical University of Kosice, Kosice 04121, Slovak Republic; peter.blistan@tuke.sk

The authors wish to make the following correction to their paper [1]. Mr. Jakub Hospodka has requested that he be removed from the list of contributing authors as he collaborated only as the authors’ project partner. The authors, therefore, wish to replace:

Miroslav Kelemen ^1,^*, Volodymyr Polishchuk ^2^, Beáta Gavurová ^3^, Stanislav Szabo ^4^, Róbert Rozenberg ^4^, Martin Gera ^5^, Jaroslaw Kozuba ^6^, Jakub Hospodka ^7^, Rudolf Andoga ^4^, Adriana Divoková ^8^ and Peter Blišťan ^9^

^1^ Faculty of Aeronautics, Technical University of Kosice, Kosice 04121, Slovak Republic

^2^ Faculty of Information Technologies, Uzhhorod National University, Uzhhorod 88000, Ukraine; volodymyr.polishchuk@uzhnu.edu.ua

^3^ Research and Innovation Centre Bioinformatics, USP TECHNICOM, Technical University of Košice, Kosice 04001, Slovak Republic; beata.gavurova@tuke.sk

^4^ Faculty of Aeronautics, Technical University of Kosice, Kosice 04121, Slovak Republic; stanislav.szabo@tuke.sk (S.S.); robert.rozenberg@tuke.sk (R.R.); rudolf.andoga@tuke.sk (R.A.)

^5^ Faculty of Mathematics, Physics and Informatics, Comenius University in Bratislava, Bratislava, Mlynská Dolina 84248, Slovak Republic; mgera@fmph.uniba.sk

^6^ Faculty of Transport, Silesian University of Technology, Gliwice 44100, Poland; jaroslaw.kozuba@polsl.pl

^7^ Faculty of Transport, Czech Technical University in Praque, Praque 16000, Czech Republic; xhospodka@fd.cvut.cz

^8^ Faculty of Mechanical Engineering, Technical University of Kosice, Kosice 04121, Slovak Republic; adadivokova@gmail.com

^9^ Faculty of Mining, Ecology, Process Control and Geotechnology, Technical University of Kosice, Kosice 04121, Slovak Republic; peter.blistan@tuke.sk

with:

**Miroslav Kelemen ^1^, Volodymyr Polishchuk ^2^, Beáta Gavurová ^3^, Stanislav Szabo ^1^, Róbert Rozenberg ^1^, Martin Gera ^4^, Jaroslaw Kozuba ^5^, Rudolf Andoga ^1^, Adriana Divoková ^6^ and Peter Blišťan ^7^**

^1^ Faculty of Aeronautics, Technical University of Kosice, Kosice 04121, Slovak Republic; stanislav.szabo@tuke.sk (S.S.); robert.rozenberg@tuke.sk (R.R.); rudolf.andoga@tuke.sk (R.A.)

^2^ Faculty of Information Technologies, Uzhhorod National University, Uzhhorod 88000, Ukraine; beata.gavurova@tuke.sk

^3^ Research and Innovation Centre Bioinformatics, USP TECHNICOM, Technical University of Košice, Kosice 04001, Slovak Republic; beata.gavurova@tuke.sk

^4^ Faculty of Mathematics, Physics and Informatics, Comenius University, Bratislava, Mlynská Dolina 84248, Slovak Republic; mgera@fmph.uniba.sk

^5^ Faculty of Transport, Silesian University of Technology, Gliwice 44100, Poland; jaroslaw.kozuba@polsl.pl

^6^ Faculty of Mechanical Engineering, Technical University of Kosice, Kosice 04121, Slovak Republic; adadivokova@gmail.com

^7^ Faculty of Mining, Ecology, Process Control and Geotechnology, Technical University of Kosice, Kosice 04121, Slovak Republic; peter.blistan@tuke.sk
